# RATES OF FITNESS DECLINE AND REBOUND SUGGEST PERVASIVE EPISTASIS

**DOI:** 10.1111/evo.12234

**Published:** 2013-09-02

**Authors:** L Perfeito, A Sousa, T Bataillon, I Gordo

**Affiliations:** 1 Instituto Gulbenkian de CiênciaOeiras, Portugal; 3 Bioinformatics Research Center, Aarhus University8000C Aarhus, Denmark

**Keywords:** Epistasis, fisher geometrical model, fitness recovery, mutation accumulation, rate of adaptation

## Abstract

Unraveling the factors that determine the rate of adaptation is a major question in evolutionary biology. One key parameter is the effect of a new mutation on fitness, which invariably depends on the environment and genetic background. The fate of a mutation also depends on population size, which determines the amount of drift it will experience. Here, we manipulate both population size and genotype composition and follow adaptation of 23 distinct *Escherichia coli* genotypes. These have previously accumulated mutations under intense genetic drift and encompass a substantial fitness variation. A simple rule is uncovered: the net fitness change is negatively correlated with the fitness of the genotype in which new mutations appear—a signature of epistasis. We find that Fisher's geometrical model can account for the observed patterns of fitness change and infer the parameters of this model that best fit the data, using Approximate Bayesian Computation. We estimate a genomic mutation rate of 0.01 per generation for fitness altering mutations, albeit with a large confidence interval, a mean fitness effect of mutations of −0.01, and an effective number of traits nine in *mut*S^−^ *E. coli*. This framework can be extended to confront a broader range of models with data and test different classes of fitness landscape models.

The distribution of effects of new mutations and how their interactions cause changes in fitness are fundamental quantities in our understanding of evolution. It is generally acknowledged that the genetic background has a strong influence on the phenotypic effect of mutations (De Visser et al. 2011). If the phenotype is relevant for fitness, natural selection will act differently on the mutation depending on its genomic background. Throughout this article, a mutation is said to be epistatic if its fitness effect, and thus ultimately its fate in the population, depends on the genetic background where it appears.

Numerous population genetics models have been built assuming that mutations affect fitness independently, that is, epistasis is absent. In the most classical experiment designed to determine the distribution of fitness effects of mutations, epistasis is typically ignored. In this type of experiment, populations are propagated at the lowest possible population size, minimizing the effect of natural selection and allowing deleterious mutations to accumulate close to the rate at which they appear (Bateman 1959; Mukai 1964). Interestingly, the results of these mutation accumulation (MA) experiments have been continuously reanalyzed using different methods (Bataillon 2000; Halligan and Keightley 2009), but usually under the same model. The rate and mean effect of mutations are assumed constant and independent of the current fitness of the genotype hit by a new mutation. However, empirical work suggests that epistasis for fitness might be pervasive, with positive or negative mean depending on the organism studied (Sanjuán and Elena 2006). Many studies are focused on deleterious mutations, as these are the most common. However recent experiments, focusing on discrete sets of beneficial mutations underlying fitness changes observed in experimental populations, support the notion that beneficial mutations may generally interact in an antagonistic way in microbes (Chou et al. 2011; Khan et al. 2011; Rokyta et al. 2011). Other studies show mean levels of epistasis ranging from antagonistic to nearly neutral in bacteria (Trindade et al. 2009; Ward et al. 2009).

Understanding the type of epistasis underlying fitness has important consequences for a number of evolutionary questions. For example, it is expected that sexual reproduction will be favored if most deleterious mutations interact synergistically (Kondrashov 1988). One important type of epistasis is sign epistasis, in which a mutation is deleterious or beneficial depending on the background. If it is common, then the number of paths that can be taken during adaptation might be severely constrained (Weinreich et al. 2005). The pattern of epistasis is often thought to be specific to a given species. However, recurrent observations suggest an interesting empirical rule: genotypes with lower fitness in a given environment adapt faster to that environment. This is observed across very diverse organisms: phage (Silander et al. 2007), *Escherichia coli* (Moore and Woods 2006; Khan et al. 2011), *Saccharomyces cerevisiae* (Murphy and Zeyl 2012), *Aspergillus nidulans* (Gifford et al. 2011), and *Caenorhabditis elegans* (Estes and Lynch 2003). All of these studies are focused on the fitness effects of beneficial mutations in diverse backgrounds. An open question is whether the same rule applies to other types of mutations.

Many experiments, particularly those aimed at studying adaptation, can only detect the effects of mutations that survive genetic drift. Because the probability of escaping stochastic loss depends itself on the selection coefficient of mutations, estimating how fitness effects depend on genetic background requires taking into account the effect of natural selection. Three main factors affect the efficiency of selection and thus the probability of empirically detecting a beneficial mutation: population size, spatial structure, and recombination. In small or fragmented populations, selection will be relatively inefficient both at purging deleterious and at fixing beneficial mutations. In very large populations, deleterious mutations will have little chance of fixing, whereas beneficial mutations enjoy a fixation probability directly proportional to their effect on fitness (Haldane 1927), assuming weak selection. In the absence of recombination, the efficiency of selection is also reduced because mutations are locked together in the same genotype. For example, if a beneficial mutation appears in a background that already harbors a deleterious mutation, it will only have a good chance of fixing if its effect outweighs the deleterious effect of the first mutation (Charlesworth et al. 1993; Bachtrog and Gordo 2004). In the same manner, if two beneficial mutations appear in a population on different genetic backgrounds, they will compete for fixation and one will be lost (Gerrish and Lenski 1998; Gerrish et al. 2007). This interference also occurs in the presence of small amounts of recombination (Barton 1995).

Here we analyze the results of two laboratory experiments, where population size is manipulated to modulate the efficiency of natural selection. Specifically, we reanalyze the results of an MA experiment using a strain of *E. coli* that exhibits a mutator phenotype (*mut*S^−^). In this experiment *E. coli* evolved under drastic periodic bottlenecks consisting of a single cell. We also complement this MA study with a readaptation experiment. In this experiment several independent genotypes, stemming from the MA experiment, are allowed to evolve under large effective population size. We explore how fitness changes (decreases in small populations and increases in large populations) depend on the genetic background. We show that the fitness change is strongly correlated with initial fitness in both experiments, suggesting that epistasis is very common.

Not accounting for epistasis fails to uncover the salient features of the fitness trajectories observed in each experiment. Therefore, we should consider models where epistasis is pervasive. One widely used phenotype-based model of evolution is Fisher's geometrical model, hereafter referred to as FGM (Fisher 1930). This model has been used to interpret patterns of adaptation in large populations and to derive theoretical distributions of fitness effects of mutations and epistasis (Martin et al. 2007). It has also been invoked to help understanding the evolutionary trajectory of small populations (Tenaillon et al. 2007). So far, it has rarely been used to explain the fitness decay typically observed during evolution of very small populations, in particular of MA experiments (but see Poon and Otto 2000; Martin and Lenormand 2006). Under FGM, one can derive the distribution of fitness effects, *s*, of a single mutation depending on the current background and pairwise epistasis <ε> between random mutations (Martin et al. 2007). The first is expected to follow a shifted gamma distribution with a given mean, *E*(*s*), and the latter is predicted to be a normal distribution with mean *E*<ε> = 0. Both *E*(*s*) and <ε> do not depend on the genetic background, although the variances do in the simplest version of FGM. Other versions of the FGM where <ε> is nonzero have also been proposed (Gros et al. 2009). The simplest model pictures evolution as a walk in a geometric space of *n* phenotypic traits. It assumes that, within a given constant environment, a single value for each of the *n* traits is optimal, with fitness decreasing smoothly for phenotypes away from that optimum. When a population is located at the optimum, any random mutation, irrespective of its size or direction, will cause a deviation from the optimum and so will necessarily be deleterious. FGM incorporates naturally a dependence of the rate of evolution on the initial phenotype. Importantly, this model can account for several empirical patterns (Tenaillon et al. 2007; Gordo and Campos 2012) including the fitness effects of mutations (Schoustra et al. 2009; Bataillon et al. 2011; Sousa et al. 2012; Trindade et al. 2012) and pairwise epistasis (Martin et al. 2007). Here, we use FGM to analyze the results of the measured fitness change, under both small and large population sizes. We introduce a statistical framework that allows combining information from both experiments to estimate the parameters underlying this model. We show that patterns of fitness change in both experiments can be quantitatively captured under FGM.

## Materials and Methods

### BACTERIAL STRAINS AND GROWTH CONDITIONS

The strains and growth conditions used in this study were as described in Trindade et al. (2010). Briefly, for the MA experiment and its analysis, the two strains used were *E. coli* K12 MG1655 srl::Tn10 *mutS* and K12 MG1655 srl::Tn10 Δ*ara mutS*. The first strain was the ancestor for the MA experiment, and the second was used as the reference strain for the competition assays in the fitness measurements. The deletion in the arabinose operon is a phenotypic marker that allows distinguishing the two strains in competition, because these mutants give rise to red colonies when plated in Tetrazolium agar (TA; Lenski et al. 1991).

For the recovery experiment, 23 clones derived from the MA lines were studied. These clones cover a range of initial fitnesses from 0.77 to 0.99 and include 16 clones from the 50th bottleneck, six clones from the 20th bottleneck, and one clone from the 70th bottleneck.

To allow the clones to readapt, evolution under large population sizes was performed. In the first day, each experimental line was started with 10 mL of LB medium seeded with ∼1.5 × 10^4^ individuals and incubated at 37°C with agitation for 24 h. This period allowed for ∼20 generations after which appropriate dilutions were made from each culture and another ∼1.5 × 10^4^ individuals were used to seed the next passage. This procedure was repeated for ∼240 generations after which fitness from all the lines was measured. One of the clones was propagated in three independent replicates and five were propagated in two replicates. The remaining clones were propagated once, yielding in total 30 adapted populations.

### MUTATION RATE AND FITNESS ESTIMATION

Because it is possible for the mutation rate to evolve, we looked for a phenotypic signature of a change in mutation rate, by estimating the frequency of Rifampicin resistance with a fluctuation assay. We tested 34 lines, these comprised the 23 lines used as the ancestors of the recovery experiment and 11 lines after the recovery experiment (Fig. S1).

The fitness assays of the MA lines are described in Trindade et al. (2010). Fitness of the recovery lines (after readaptation for 120 and 240 generations) was assayed in the same way as the MA lines. Briefly, competitor and reference strains were mixed in a proportion of 1:1 and used to seed 10 mL of LB. The competition tube was then incubated for 24 h at 37°C with agitation. Appropriate dilutions, of both initial and final bacterial populations, were plated in TA to access the ratio between competitor and reference strains.

Selective coefficients were estimated as the difference in growth between the test strain and the reference strain, normalized by the number of generations elapsed for the reference strain (Chevin 2011). For each competition replicate, the difference in growth between the test strain (*a*) and the reference strain (*b*) is given by:


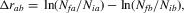


where *N_fa_* and *N_fb_* are the number of test and reference bacteria, respectively, after the competition, and *N_ia_* and *N_ib_* are the number of test and reference bacteria, respectively, before the competition. The relative growth rate estimated this way refers to a 24-h growth cycle and must be normalized by the generation time of the reference to be comparable with the population genetics model we use. The number of generations (*G*) elapsed for the reference was estimated for each competition replicate, assuming no death, as follows:


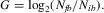


Fitness of strain *a* relative to the reference *b* (*W*_a_) is then given by:


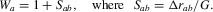


The fitness of the ancestor of the MA was measured in the same way and all fitness values were then standardized such that the fitness of this ancestor was 1. We compared two models for the relation between the decrease in fitness with increasing number of bottlenecks: a linear and a quadratic model, using the function LinearModelFit in Mathematica 8.0 (Wolfram Research, Inc. 2010).

### SIMULATIONS OF THE EVOLUTION PROCESS UNDER FGM

To simulate data sets under FGM that are directly comparable with our observed empirical data, we performed individual-based Monte Carlo simulations.

As the MA experiment involves bottlenecks of one cell every day, in the simulations of MA lines we assumed discrete generations and a demography that reflects this periodic fluctuation in population size. Accordingly, we crashed the populations to a size *N* = 1, every 23 generations. After these crashes the population expanded, doubling in size every generation, until the next crash occurred. Therefore, the harmonic mean of the population size was 23, which was taken as the effective population size *N_e_* (Wahl et al. 2002).

In our MA experiment, the founding clone was highly fit, so we expect the vast majority of new mutations to be deleterious. We started the simulations of the MA experiment with a unique genotype residing at the phenotypic multivariate optimum, i.e. with a maximum fitness of 1. Given these conditions we simulated the mutation-selection-drift process under FGM. FGM assumes that *n* phenotypic traits underlie fitness differences among individuals and each trait is under stabilizing (optimizing) selection. Without loss of generality we assumed that the optimum phenotypic value was zero for each trait. A special case of FGM was assumed by considering a Gaussian fitness landscape, whereby each individual is represented by a vector of *n* phenotypic trait values **x** = (*x*_1_, *x*_2_,…, *x_n_*) and has fitness 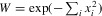. Mutations are fully pleiotropic and occur each generation. The FGM we considered here is an isotropic version of the ones in Martin and Lenormand (2006) and Waxman and Welch (2005).

We assumed that the number of new mutations in each individual was Poisson distributed with rate *U* per individual per generation. In addition, each mutation is fully pleiotropic and changes all traits (*x_i_*) and thus ultimately fitness. Each mutation therefore causes a change in each value of *x_i_*, which is normally distributed with mean 0 and variance σ^2^. Individuals are then selected to the next generation according to their fitness. In this model, the mean fitness effect of a mutation “hitting” a genotype initially residing at the multivariate phenotypic optimum is *E*(*s*) = −*n*σ^2^.

For the recovery experiment, we assumed a constant effective population size, and three different initial starting fitnesses, covering the range of variation observed in the experiment. *N_e_* was approximated by the harmonic mean of the population size *N* in the actual recovery experiment. We then let the population evolve for 240 generations and recorded the values of fitness increase after 120 and 240 generations, for each starting fitness (Fig. S2). The slope of a linear regression of the fitness change, at each of these time points, as a function of initial fitness was then measured. As these simulations, involving a large *N_e_*, are computationally very intensive, we did not follow all of the possible starting fitnesses considered in the actual experiment.

For the simulations in Figure 3, the same model was used, except only one mutation per run was recorded. The probability of each mutation surviving drift was estimated by (1 − *e*^−2*s*^)/(1 − *e*^−2*Nes*^) (Kimura 1968). The *N_e_* used was 23 for the MA simulations (Fig. 3) and 10^5^ for the fitness recovery simulations.

Finally, under the Gaussian FGM assumed here, the increase in fitness when close to the optimum is expected to be linear with initial fitness (Martin and Lenormand 2008), and we have checked this assumption with a small subset of simulations performed with many starting fitnesses (not shown).

### ESTIMATION OF PARAMETERS BY APPROXIMATE BAYESIAN COMPUTATION (ABC)

Our goal was to estimate jointly the genomic mutation rate for fitness (*U*) and the parameters underlying FGM (i.e., *n* the number of independent phenotypic traits under stabilizing selection and σ the scaled mean phenotypic effect of a mutation) from empirical data (collectively termed ***D***). Hereafter we “collect” the three parameters in a vector **θ *=*** (*U*, *n*, σ).

Empirical data available to us were of two distinct kinds: fitness measurement of MA lines at regular time intervals and fitness measurement during the fitness recovery experiment.

To estimate θ, we note that the likelihood of our data ***D*** cannot be obtained in a closed form under FGM given the complications introduced jointly by demographics and selection. However, this likelihood can be defined implicitly because we can simulate data sets fully comparable to ***D*** under FGM. We therefore used an approximate Bayesian framework where the likelihood of the full data ***D*** was approximated by replacing the likelihood of ***D*** by the likelihood of a vector of summary statistics, ***S_D_***, that summarizes our data ***D***. Note that ***S_D_*** comprises 39 statistics summarizing both the MA experiment and the fitness recovery experiment (Table 1).

**Table 1:** Overview of the set of summary statistics used for ABC inference

Summary statistic	Observed	Experiment
<0.77^1^	0	Bot 10
[0.77 – 0.81[	0	
[0.81 – 0.85[	1	
[0.85 – 0.89[	1	
[0.89 – 0.93[	3	
[0.93 – 0.97[	20	
>0.97	25	
<0.77	0	Bot 20
[0.77 – 0.81[	0	
[0.81 – 0.85[	4	
[0.85 – 0.89[	2	
[0.89 – 0.93[	12	
[0.93 – 0.97[	24	
>0.97	8	
<0.77	0	Bot 30
[0.77 – 0.81[	0	
[0.81 – 0.85[	2	
[0.85 – 0.89[	10	
[0.89 – 0.93[	10	
[0.93 – 0.97[	21	
>0.97	7	
<0.77	0	Bot 40
[0.77 – 0.81[	2	
[0.81 – 0.85[	5	
[0.85 – 0.89[	8	
[0.89 – 0.93[	16	
[0.93 – 0.97[	13	
>0.97	6	
<0.77	0	Bot 50
[0.77 – 0.81[	4	
[0.81 – 0.85[	10	
[0.85 – 0.89[	9	
[0.89 – 0.93[	15	
[0.93 – 0.97[	11	
>0.97	1	
Slope 120^2^	−0.7986	Fitness recovery experiment
Intercept 120	0.7585	
Slope 240	−0.8873	
Intercept 240	0.8647	

1 Observed distribution fitness values in the mutation accumulation experiment. Distributions at each time point (Bottleneck 10, 20, 30, 40, 50) are summarized using counts in seven classes of fitness values (all counts sum to 50 at each time point).

2 The observed slope and intercept of the regression line describing the fitness recovery (after 120 or 240 generations) as a function of initial fitness.

Rejection sampling was then used to approximate the posterior distribution of θ given ***S_D_***. To conduct rejection sampling, we simulated *M* “composite” data sets comprising data from both MA and fitness recovery lines under FGM. Each simulation generating an MA + recovery data set was “seeded” by first drawing parameters from a joint prior distribution π(θ).

We chose the following priors for our parameters: log_10_(*U*) is uniform in [−3.5, −0–5] and encompasses very broadly the range of genome wide mutation rates previously reported in *E*. *coli*; we chose a very flat exponential prior for σ (Fig. 4); and a uniform discrete distribution on [1, 30] that covers broadly the range of previously estimated values for this parameter (Martin and Lenormand 2006; Bataillon et al. 2011; Sousa et al. 2012; Trindade et al. 2012).

We implemented rejection sampling by retaining the fraction *tol* of parameters that seeded simulations that yielded the set of summary statistics closest to the vector of observed summary statistics ***S_D_***. In practice, we used *M* = 1,500,000 simulations and conducted rejection sampling by keeping the closest 1500 simulations (tol = 0.001). The set of parameters yielding these 1500 accepted data sets was used to approximate Π*(θ) the joint posterior distribution of the parameters.

We used a Monte Carlo approach to choose a tolerance level *tol* and study the statistical performance of our ABC estimators by rejection sampling. One data set was drawn at random from a pool of 10^6^ simulated data sets, rejection sampling was then performed using three tolerance levels on the remaining data sets to obtain ABC parameter estimates. Prediction error was calculated by adding mean (squared) bias and variance as 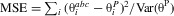, where 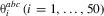 are the ABC estimates and 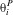 are the true values of the parameters use for simulating data.

Preliminary results based on 50 simulated data sets exploring three different tolerance levels (*tol* = 0.1%, 1%, 10%) suggested that the lowest tolerance (*tol* = 0.1%) yielded the best prediction errors for the ABC estimates (not shown). We cannot exclude that ABC estimators with even lower MSE can be devised by using alternative weighting and/or sets of summary statistics but this is beyond the scope of the present study. Moreover, we showed via simulation that at *tol* = 0.1% and with the set of summary statistics we used, ABC estimates do not err systematically (Fig. S3).

All rejection sampling, posterior approximations and Monte Carlo simulations for evaluating the performance of the ABC estimator were conducted with the statistical software *R* and using the package *abc* (Csilléry et al. 2012).

## Results

### INITIAL FITNESS AS A PREDICTOR OF RATES OF FITNESS CHANGE THROUGH TIME

#### Initial fitness predicts the decrease in fitness in populations experiencing intense genetic drift

**Figure 1:**
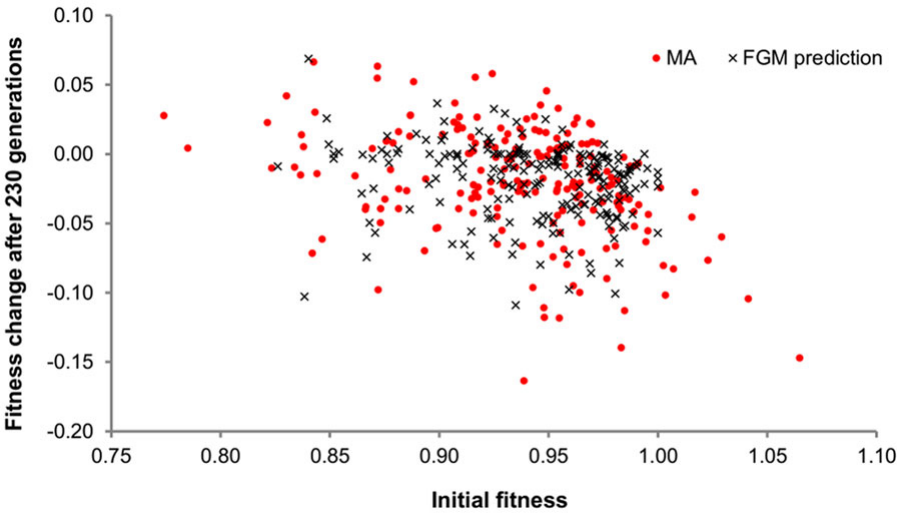
Fitness change as a function of initial fitness during the mutation accumulation (MA). The fitness change of each line over 10 bottlenecks (or 230 generations) is shown as a function of that line's fitness before that interval: the difference between the 10th and the 20th bottlenecks is shown as a function of fitness at the 10th bottleneck, the difference between the 20th and the 30th is shown as a function of fitness at the 20th, and so on. Each MA line is represented four times, once for each interval. The experimental data (shown as dots) is taken from Trindade et al. (2010). Fifty simulations of data sets under Fisher's geometrical model (FGM; shown as crosses) were performed as described in the text, using as parameters *n* = 9, *U* = 0.01, and σ = 0.034. In both cases, a significant correlation between the change in fitness and initial fitness is obtained. For the experimental data, the slope of the linear regression is −0.32 with CI (−0.43 to −0.21) and for the data under FGM the slope is −0.15 with the 95% confidence interval (−0.24 to −0.05).

We reanalyzed the data of an MA experiment in *E. coli* previously reported (Trindade et al. 2010) to test the extent to which initial fitness of genotypes can influence subsequent changes in fitness due to MA. In this experiment, 50 lines of a mutator strain were propagated by daily transfer. Each transfer consisted of a bottleneck down to a single cell (Methods in Trindade et al. 2010) and mean fitness was measured every 10 bottlenecks, that is, approximately every 230 generations. During the course of this experiment, mean fitness was reduced by 10% on average, and some of the lines reached a fitness level as low as 0.78 (relative to the ancestor). The decline in mean fitness and increase in variance between MA lines through time allowed the estimation of the deleterious mutation rate (*U_d_*) and the mean effect of deleterious mutations (*E*(*s_d_*)), using a variant of the Bateman–Mukai (BM) method, which accounts for the effects of natural selection between bottlenecks (Gordo and Dionisio 2005; Trindade et al. 2010). The inferred mutation rate was *U* = 0.005 deleterious mutations per genome per generation with a mean effect of *E*(*s_d_*) = −0.03. This inference assumes that all mutations are deleterious with identical fitness effects, and that no epistasis occurs. There are two ways in which we can look for evidence of epistasis in an MA experiment. One method that has been widely used is to examine the decrease in fitness, averaged over all MA lines, through time. If there is no directional epistasis (<ε> = 0), then the decrease in the logarithm of fitness with time is expected to be linear. Deviations from linearity indicate some level of mean epistasis, either positive or negative, depending on the curvature found (De Visser et al. 2011). For this MA, the regression coefficient of the logarithm of fitness with time was 0.98 (Akaike information Criterion [AIC] of −36.3). A quadratic model applied to this data did not improve the fit (AIC = −36.5). Hence, using this method, we have no evidence that the mean epistatic effect was different from zero. However, we note that this approach is merely regressing the mean fitness of all lines. Independent lines accumulated different numbers of mutations along time, hence the mean may not be very sensitive. As a complement, we examined the change in fitness after a period of 10 bottlenecks as a function of the initial fitness before these 10 bottlenecks. If epistatic interactions do not exist, no correlation between the fitness of a line and its decrease in fitness in subsequent bottlenecks is to be expected. Contrary to this expectation, we observed that lines with a higher fitness were more likely to show a decrease in fitness than lines with lower initial fitness. Figure 1 shows that the change in fitness and initial fitness are correlated (Spearman correlation coefficient of −0.34, *P* < 10^−7^). We conclude that the change in fitness depends strongly on the initial fitness. This points to the occurrence of widespread epistasis among spontaneous mutations affecting fitness in *E. coli*. These findings prompted us to reanalyze the data under a model that explicitly accounts for the possibility of widespread epistasis among mutations thus yielding novel estimates of the rate and effects of mutations in this MA experiment.

#### Initial fitness predicts the increase in fitness in large populations

Fitness changes observed in the course of MA experiments are generally negative (see Halligan and Keightley 2009 for a review). We sought to perform a recovery experiment where the change in fitness is expected to be positive. We also wanted to test if the rate of fitness increase depends on the genetic background. We did so by using a subset consisting of 23 MA strains covering a wide range of relative fitness (from 0.77 to 0.99) to start our fitness recovery experiment. Among these strains, 16 were obtained from the 50th bottleneck, 6 were from the 20th bottleneck, and the 23rd was obtained by propagating one of the MA lines for another 20 bottlenecks.

**Figure 2:**
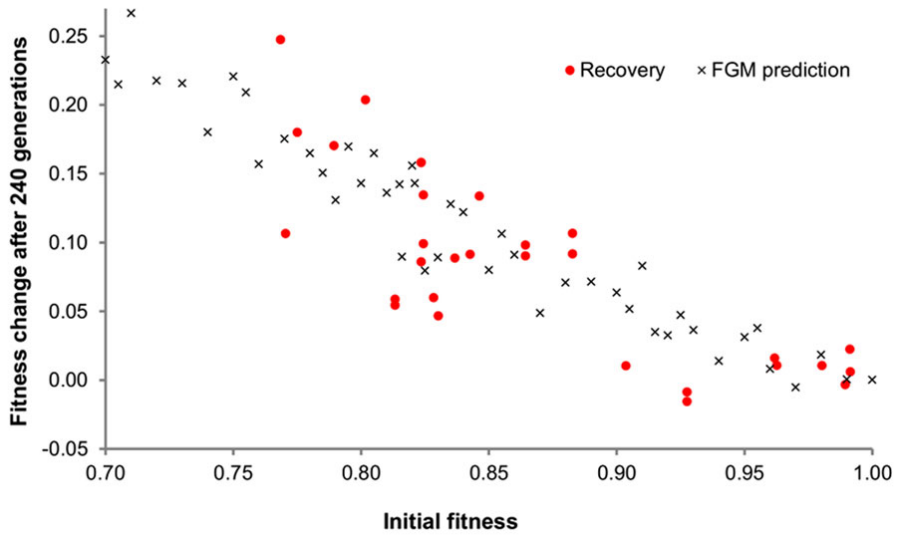
Fitness change as a function of the initial fitness. Fitness change after 12 bottlenecks (shown as dots) as a function of the initial fitness. Clones with different starting fitness were adapted for 240 generations under a large effective population size (see Methods for details). The results of Fisher's geometrical model (FGM) simulations with parameters values set to *n* = 9, *U* = 0.01, and σ = 0.034 are shown as crosses. In both cases, a significant correlation between the change in fitness and initial fitness is obtained. For the experimental data, the slope of the linear regression is −0.77 with CI (−0.97 to −0.57) and for the data under FGM the slope is −0.84 with the 95% confidence interval (−0.86, −0.82).

We adapted these clones to the medium where the competitions were performed, during 240 generations, under serial dilution thereby imposing an effective population size (*N_e_*) of 3 × 10^5^. We then measured fitness in the same conditions used for measuring fitness of the MA lines. Figure 2 shows the change in mean population fitness as a function of the fitness of the founding clone. Clearly, the rate of adaptation depends on the genetic background (Spearman correlation coefficient of −0.76, *P* < 10^−5^). In fact, the lower the initial fitness of the clone, the higher its fitness increase. This relationship was observed for the clones sampled from the MA at the 20th and at the 50th bottleneck, as shown in Figure S4. In this experiment, 63% of the variance in fitness could be predicted by the initial fitness, and only two lines exhibited no increase in fitness during the 240 generations (Fig. 2).

Although not impossible, back mutation is an unlikely explanation for these results, given the estimates of point mutation rates in *E. coli* (Drake 1991; Wielgoss et al. 2011). Compensatory mutations happening at other sites throughout the genome are, however, expected to be far more common (Poon et al. 2005), although studies addressing their rate and distribution remain rare, with most studies examining compensation in the context of antibiotic resistance (Schrag et al. 1997; Levin et al. 2000; Sousa et al. 2012).

### FISHER GEOMETRICAL MODEL PREDICTS THE PATTERNS OBSERVED IN THE MA AND IN THE RECOVERY EXPERIMENT

#### The patterns of fitness change expected under FGM

The data of both the MA and the recovery experiment show a clear dependence of fitness change on initial fitness (*W*_0_) as illustrated in Figures 1 and 2. This pattern has been very rarely reported in previous MA experiments (see Bataillon 2000; Halligan and Keightley 2009 for a review). We note however that under FGM, a fitness landscape model that has received a lot of theoretical attention, the distribution of effects of incoming mutations on a genotype, changes drastically as a function of its current fitness relative to the optimum. In particular, under FGM, we expect that in well-adapted populations new mutations will be mostly deleterious, whereas in poorly adapted populations a sizeable fraction of the mutations will be beneficial (compensatory).

**Figure 3:**
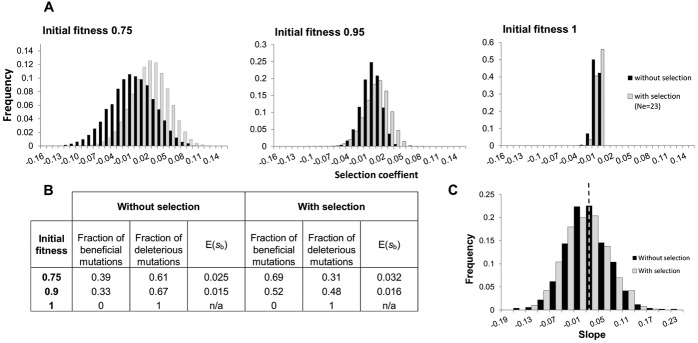
Distribution of fitness effects of mutations before and after selection. (A) The distribution of spontaneous mutations was generated by 1000 Monte Carlo simulations of Fisher's geometrical model (FGM) as described in the text, using as parameters *n* = 9, *U* = 0.01, and σ = 0.034. The distribution of mutations that escape genetic drift was estimated as the fraction of spontaneous mutations that escape drift in a population of effective size of 23 individuals, corresponding to the population expansion occurring in the MA (Gordo and Dionisio 2005). (B) Mean of the distributions in (A), considering different starting fitnesses and fraction of beneficial and deleterious mutations. (C) Distribution of slopes of the linear regression of change in fitness with initial fitness under FGM with no selection (black bars, mean = −0.003) and with selection (gray bars, mean = −0.015) in the MA lines. The dashed line shows the location of slope 0. Parameter values used are the same as in (A).

We compared the predictions of FGM to the experimental observations, taking into account the demographic parameters used. Namely, we modeled the extreme fluctuations in population size in the MA, and allowed selection to occur during the growth of a line before it is bottlenecked (see Methods). When simulating FGM under these conditions, we observed than even with the drastic bottleneck procedure, the distribution of mutations accumulated is skewed by selection for beneficial and small effect deleterious mutations (Fig. 3A and B). Incorporating selection in the MA lines, FGM could reproduce the patterns seen in the experimental data, both in the MA (Fig. 1) and fitness recovery (Fig. 2). To evaluate the importance of selection occurring during the MA, we simulated MA data sets using FGM with the parameters that best fit the data (see the next section for the ABC approach we used to estimate them). In these simulations, mutations drawn from FGM accumulated with or without selection and we calculated the slopes of the linear regression of the change in fitness with starting fitness. Without selection the distribution of slopes was centered on zero with a small range of negative or positive values, whereas for MA data simulated with selection an average negative slope was observed (Fig. 3C).

The linear relation of change in fitness with starting fitness appears to capture well the pattern of the recovery experiment. Under FGM, and assuming a Gaussian fitness landscape, Martin and Lenormand (2008) predicted a linear relation between the distance to the peak (*s*_0_) and the mean effect of beneficial mutations fixed during the first step of adaptation (i.e., *E*(sb|fix) = 4*s*_0_/(4 + *n*)). This prediction is valid when the starting genotype is close to the optimum and ignoring clonal interference. If we take the data for the clones with highest starting fitness (*W_i_* > 0.85), those that start closer to the optimum and those for which clonal interference is least expected to occur, and if we further assume that the fitness increase observed (DW) results from the first step of adaptation, we can calculate the slope of the linear regression of DW on *s*_0_ ∼ 1 − *W_i_*, and get a crude estimate of *n*. Under these assumptions *n* is estimated to be around 3.

#### Estimating parameters of FGM from experimental data

Given the capacity of FGM to reproduce the pattern present in the data (Figs. 1, 2), we next sought to estimate jointly the parameters of this model (*n* and *σ*, see Methods for details) as well as the genomic mutation rate (*U*) from our experimental data. To do so, we used an ABC framework where we combined empirical data from both experiments using several summary statistics (Table 1). These statistics describe the fitness distribution along bottlenecks of the MA experiment and the fitness change in two time points of the recovery experiment (see Methods). We then confronted these empirically observed summary statistics with those predicted under FGM given a value of *U*, *n*, and *σ*. ABC approaches have been very popular when analyzing patterns of nucleotide diversity under various demographic models (Robert et al. 2011). However, to our knowledge, they have never been used systematically to analyze these types of experiments. Previous studies relied on Monte Carlo simulations of data sets which were used to “fit” data from MA experiments with EMS mutagenesis (Davies et al. 1999) or to estimate fitness effect of two interfering beneficial mutations from patterns of adaptation over time in experimental populations (Hegreness 2006).

Here, adopting an ABC approach allowed us to obtain approximate posterior distributions for the genome-wide mutation rate and parameters of the FGM model in a case where an explicit likelihood function cannot be obtained in a closed form for each experiment due to the need to consider the joint effect of rather complicated demographics and selection. Our approach also allowed us to integrate data from both experiments (but see also Fig. S5 for posterior distribution of the parameters inferred separately from each experiment). We first evaluated the performance of our ABC estimator when considering data generated under the FGM model itself. Using sets of simulated data sets under FGM that mimic our data, we show that our ABC framework yields accurate estimates of genomic mutation rates and the FGM landscape parameters (Fig. S3).

**Figure 4:**
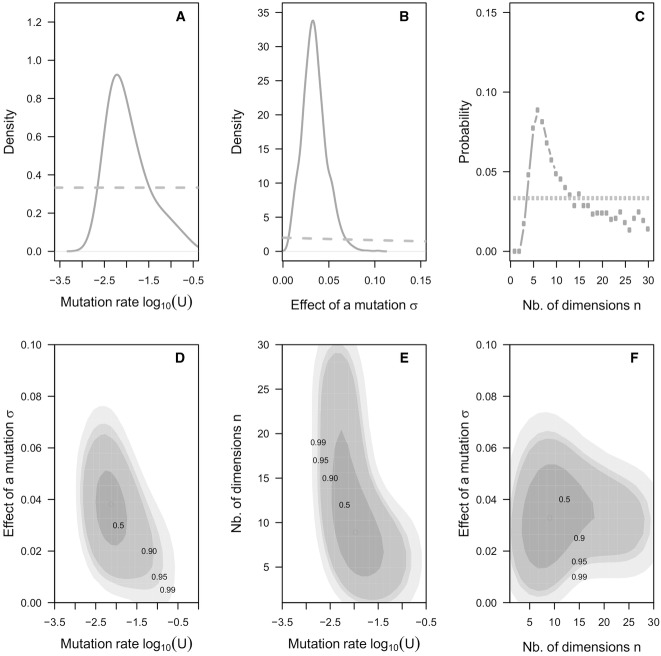
Approximate posterior densities for parameters of Fisher's geometrical model. All posterior densities are approximated using a set 1000 accepted simulations (tolerance rate of 0.001, see Methods for details). Prior densities are in orange. Marginal posteriors are in blue and regions of highest posterior densities are in gray. (A) Prior and approximate marginal posterior for the genome wide mutation rate for fitness (*U*). Note the log_10_ scale. (B) Prior and approximate marginal posterior for the scaled phenotypic effect of mutations, σ. (C) Prior and approximate posterior probability distributions for the number of dimensions, *n*. Note that these are discrete probability distributions. (D) Bivariate approximate posterior densities for *U* and σ. A circle depicts the location of the region with highest posterior density. The regions comprising 0.5, 0.9, 0.95, and 0.99 of the probability mass of the posterior density are depicted using gray shadings. (E) Bivariate approximate posterior densities for *U* and *n*. Legend as in D. Note that for graphical convenience the joint posterior distribution over *n*, and *U* was smoothed by “jittering” the values of *n* to make them continuous. (F) Bivariate approximate posterior densities for σ and *n*. Legend as in D. Note that for graphical convenience the joint posterior distribution over *n*, and σ was smoothed by jittering the values of *n* to make them apparently continuous.

Figure 4 shows the approximate posterior distributions obtained by our ABC method. Our estimates (median and 95% central posterior intervals) are *U*= 0.01[0.003 − 0.19], *n = *9[4 − 20], and σ = 0.034[0.01, 0.065]. The latter parameter estimate imply that the mean effect of single mutations on fitness is *E*(*s*) = −*n*σ^2^ = −9 × 0.034^2 ^= −0.01. This estimate of *U* is close to the one obtained using the BM method, and note that the posterior suggest substantial sampling variance. The difficulties in obtaining accurate estimates of *U* are well known for different methods (Keightley 2004). Our new estimate of the mean effect of mutations on fitness is smaller than previous BM estimate. This is not surprising given that it is well known that the BM method overestimates the mean selection coefficient of mutations when the variance of these effects is significant (Keightley and Eyre-Walker 1999; Halligan and Keightley 2009; Trindade et al. 2010).

## Discussion

We reanalyzed jointly a traditional MA experiment and a fitness recovery experiment using genotypes resulting from the MA. The patterns of fitness degeneration in MA and subsequent recovery are not new and have been reported in other organisms (Estes and Lynch 2003). What is new here is the fact that our analysis of the data also provides strong evidence for the pervasive role of epistasis among mutations underlying fitness and lends support to the idea that fitness effects of mutations vary substantially, that is the variance in mutational fitness effects is large.

Our argument relies first on the observation that patterns of temporal fitness change in both experiments are strongly dependent on initial fitness levels. We claim that this qualitative pattern of strong genetic background dependence in the data can hardly be explained without invoking the existence of epistasis. A plausible alternative explanation to the trend we observe in Figure 1 could be that the MA lines mutation rate went down during the experiment. For example, if the strains with lower fitness acquired mutations that compensated their mutator phenotype, this might explain their slower fitness decrease (but not the increases that are obvious in Fig. 2). To investigate this possibility, we used a fluctuation assay for rifampicin resistance (rif^R^) as a crude proxy for measuring genome-wide mutation rates in a subset of 34 lines. Interestingly, the set of lines assayed exhibit overall frequency of rif^R^ roughly six times lower than the frequency of the ancestral, and in some lines the decrease in frequency is more than 100-fold (Fig. S1). Although our measurements are not precise enough to compare with confidence a particular line with the ancestor, the data as a whole reject strongly the hypothesis that mutation rates have not evolved (only four lines of 34 we assayed have estimated rif^R^ higher than the ancestor, binomial test, *P* < 0.00001). Note also that our data—although preliminary—suggest no differences between MA lines and recovery lines (compare black and blue points in Fig. S1), suggesting that mutation rate evolved primarily during the MA experiment. We then examined whether there was any correlation between mutation rate estimates in the subset of MA lines and the change in fitness in the 10 bottlenecks before the estimate. We observed no correlation (*P* > 0.8). Furthermore, no correlation was found when considering the mutation rate after the recovery with the fitness increase over 240 generations (*P* > 0.2). We conclude that a change in mutation rate likely occurred in several lines but contributed little to the pattern observed in Figure 1.

Second, we note that the fitness effects of new mutations theoretically expected under FGM exhibit the salient features we empirically observed (Figs. 1, 2). This motivates the use of FGM as a plausible framework for model-based statistical inference in experimental evolution. FGM has been recently used to derive quantitative predictions for the marginal distribution of fitness effects of single mutations and/or the expected patterns of epistasis among pairs of mutations (Martin et al. 2007; Martin and Lenormand 2008; Bataillon et al. 2011; Rokyta et al 2011). These predictions have been confronted with a few data sets. In particular, the distribution of pairwise epistasis between deleterious mutations, in *E. coli*, has been found to follow a normal distribution with mean zero, following the expectation of this simple model. FGM currently stands as an attractive alternative to statistical heuristics based on extreme value theory (Rokyta et al. 2008; Bataillon et al. 2011). Here we go one step further and demonstrate that parameters underlying FGM can also be estimated jointly with genome wide mutation rate from data on temporal fitness changes in either MA or adaptation experiments.

Most of the adaptation detected in the recovery lines is likely due to compensatory mutations. Compensatory mutations are epistatic by definition: they are only beneficial in a specific background as they allow a mutant strain to revert to the ancestral phenotype but do not occur at the same nucleotide (Nagaev et al. 2001). They contribute to adaptation by increasing the number of possible beneficial mutations as fitness decreases. In addition to strictly compensatory mutations, it is possible that the ancestral background was not fully adapted to the medium where the experiment took place. In that case, there can also be new mutations, which are beneficial irrespective of the background. It is possible that both changes in the beneficial mutation rate (*U_b_*) and in the mean effect of beneficial mutations (*s_b_*) are contributing to the pattern observed in Figure 2. As was the case of the MA, our readaptation experiment supports the view that there is widespread epistasis between spontaneous mutations in *E. coli*. Empirical evidence that the distribution of fitness effects of compensatory mutations depends on the genetic background has been obtained in several studies examining the cost of antibiotic resistance (MacLean et al. 2010; Gordo et al. 2011). Some of us have recently used FGM in this context and have found that it could provide an accurate quantitative description of experimental data on the effects of these mutations (Sousa et al. 2012; Trindade et al. 2012). However, FGM has not been used to jointly study adaptation of different genotypes, which differ in mutations scattered throughout the whole genome. This is an important test, because it contributes to the establishment of general patterns.

Using FGM, one can derive how the rate and effects of both beneficial and deleterious mutations change along the evolutionary process, that is, as we walk along the single-peaked fitness landscape parameterized by the experimental data. Figure 3 illustrates our quantitative predictions, which can be challenged further by performing jointly several MA experiments and many replicate adaptation experiments, starting from distinct initial finesses (see for instance Gifford et al. 2011), a task that is beyond the scope of this article. One salient qualitative feature of the distribution of mutations under FGM is that although the mean effect does not depend on the genetic background, the variance does. In particular, as fitness goes down, the variance of the distribution of new mutations increases, with more strongly deleterious and strongly beneficial mutations. Because of that, the mean effect of mutations that survive genetic drift is predicted to depend strongly on initial fitness (Fig. 3).

Our estimate of the genomic mutation rate toward fitness altering mutations in a *mutS*^−^ *E. coli* is 0.01 per generation. Considering that this mutator has a 60-fold increase in mutation rate over the wild type, this estimate is similar to previous ones (Kibota and Lynch 1996; Trindade et al. 2010) but note that the posterior interval we report *U*[0.003 − 0.19] is very broad.

Our estimate of the mean selection coefficient of a mutation, *E*(*s*) = −0.01, lies below our previous one (Trindade et al. 2010). This is not surprising because our estimate is based on a model that intrinsically assumes a distribution of fitness effects with a variance larger than zero and explicitly accounts for compensatory mutations. This new estimate is close to that of Kibota and Lynch (1996).

Finally, we have estimated a relatively small number of traits (*n* = 9) under natural selection, which implies a low level of phenotypic complexity in this environment. This estimate is close to comparable measures of phenotypic complexity reported in other bacterial experiments (Bataillon et al. 2011; Trindade et al. 2012) but clearly more work is needed in that area and having a single common model to estimate *n* would help comparisons.

We have emphasized that FGM is an attractive model that naturally incorporates a number of biological important components: epistasis, various degrees of phenotypic complexity underlying fitness, pleiotropic effects of mutations, etc. We have also shown that FGM can account for patterns of fitness decline and recovery observed in empirical data. That being said, a number of alternative fitness models exists. One natural extension of our work is to consider a broader range of FGM-based models featuring different forms of pleiotropy (Lourenço et al. 2011), different patterns of epistasis (Gros et al. 2009), and/or different types of fixed, moving or “shaking” fitness optima (Kopp and Hermisson 2009; Gordo and Campos 2012). Another avenue is to examine radically different fitness landscape models, such as the mutational landscape model (Gillespie JH 1984), seascape model (Mustonen and Lässig 2009), or the recently proposed stickbreaking model (Nagel et al. 2012) and put these in competition with the various versions of FGM for explaining patterns of adaptation. We have shown that our ABC approach, although it is rather brute-force and relies on a large number of simulations, yields fairly accurate parameter estimates of the classical FGM (Fig. S3) and this framework can be extended to encompass a variety of models. Although model selection under ABC requires caution (see Robert et al. 2011), the methodology is naturally prone toward such an analysis. Our approach can also be a fruitful starting point to devise novel frameworks for the analysis of experimental evolution empirical data sets that borrow strength from both temporal patterns of fitness changes and snapshots of the genome at various time points of the experiments.

## Supporting Information

Disclaimer: Supplementary materials have been peer-reviewed but not copyedited.

Figure S1 Frequency of bacteria resistant to of rifampicin (100 μg/mL) in mutation accumulation and recovery lines.

Figure S2 Change in fitness after 120 (red symbols) and 240 generations of adaptation (black symbols).

Figure S3 Distribution of ABC estimates for data simulated under FGM and known genomic mutation rates.

Figure S4 Fitness changes as a function of initial fitness for two points in the mutation accumulation experiment.

Figure S5 Posterior distributions obtained by fitting separately mutation accumulation and fitness recovery data sets.
