# Supplementary figures and images for: RATES OF FITNESS DECLINE AND REBOUND SUGGEST PERVASIVE EPISTASIS
Source: Evolution. 2013 Sep 2;68(1):150–62. doi: 10.1111/evo.12234 (PMC3912910; doi:10.1111/evo.12234)

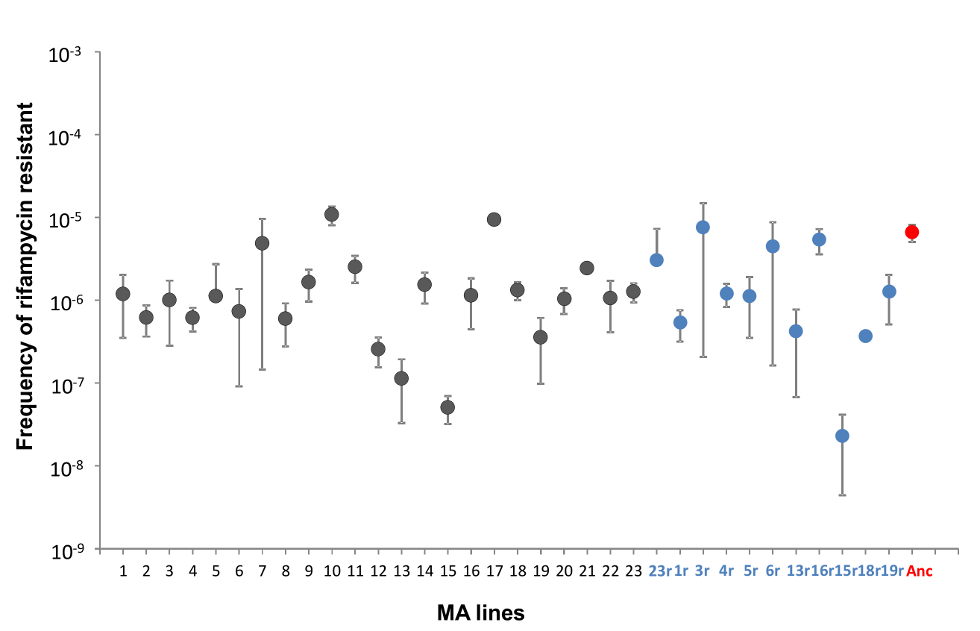

Supplement: Figure S1 — Frequency of bacteria resistant to of rifampicin (100 μg/mL) in mutation accumulation and recovery lines. [file evo0068-0150-sd1.tif]

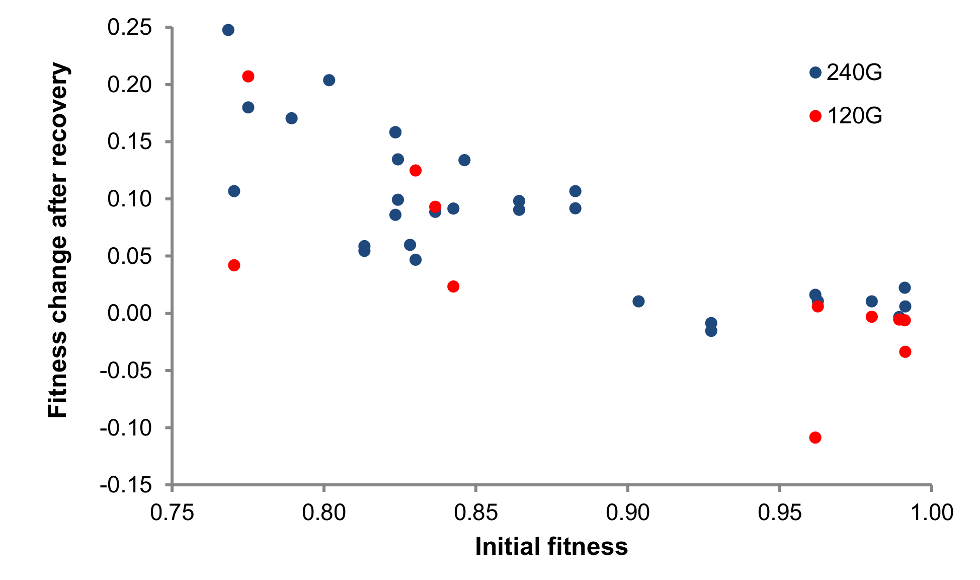

Supplement: Figure S2 — Change in fitness after 120 (red symbols) and 240 generations of adaptation (black symbols). [file evo0068-0150-sd2.tif]

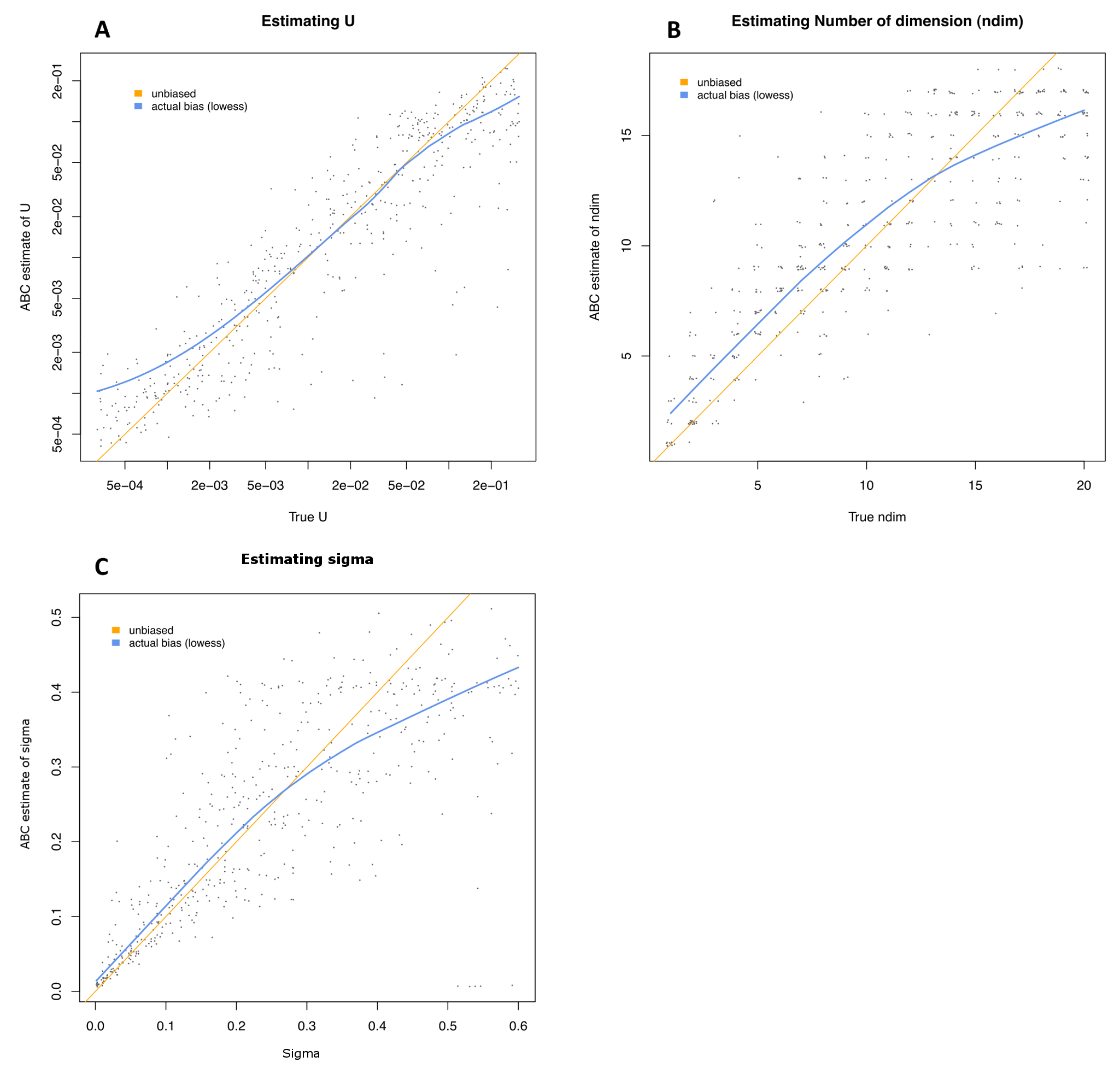

Supplement: Figure S3 — Distribution of ABC estimates for data simulated under FGM and known genomic mutation rates. [file evo0068-0150-sd3.tif]

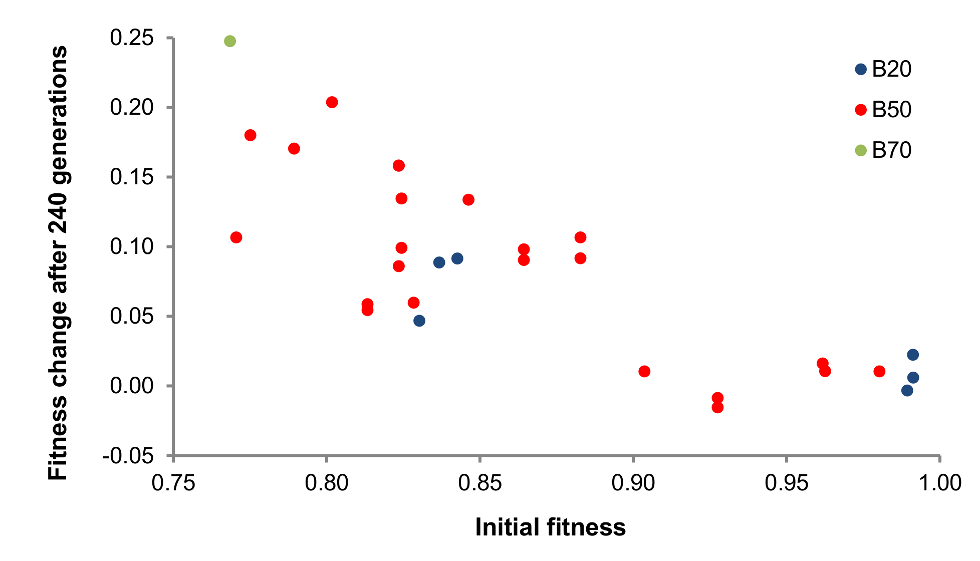

Supplement: Figure S4 — Fitness changes as a function of initial fitness for two points in the mutation accumulation experiment. [file evo0068-0150-sd4.tif]

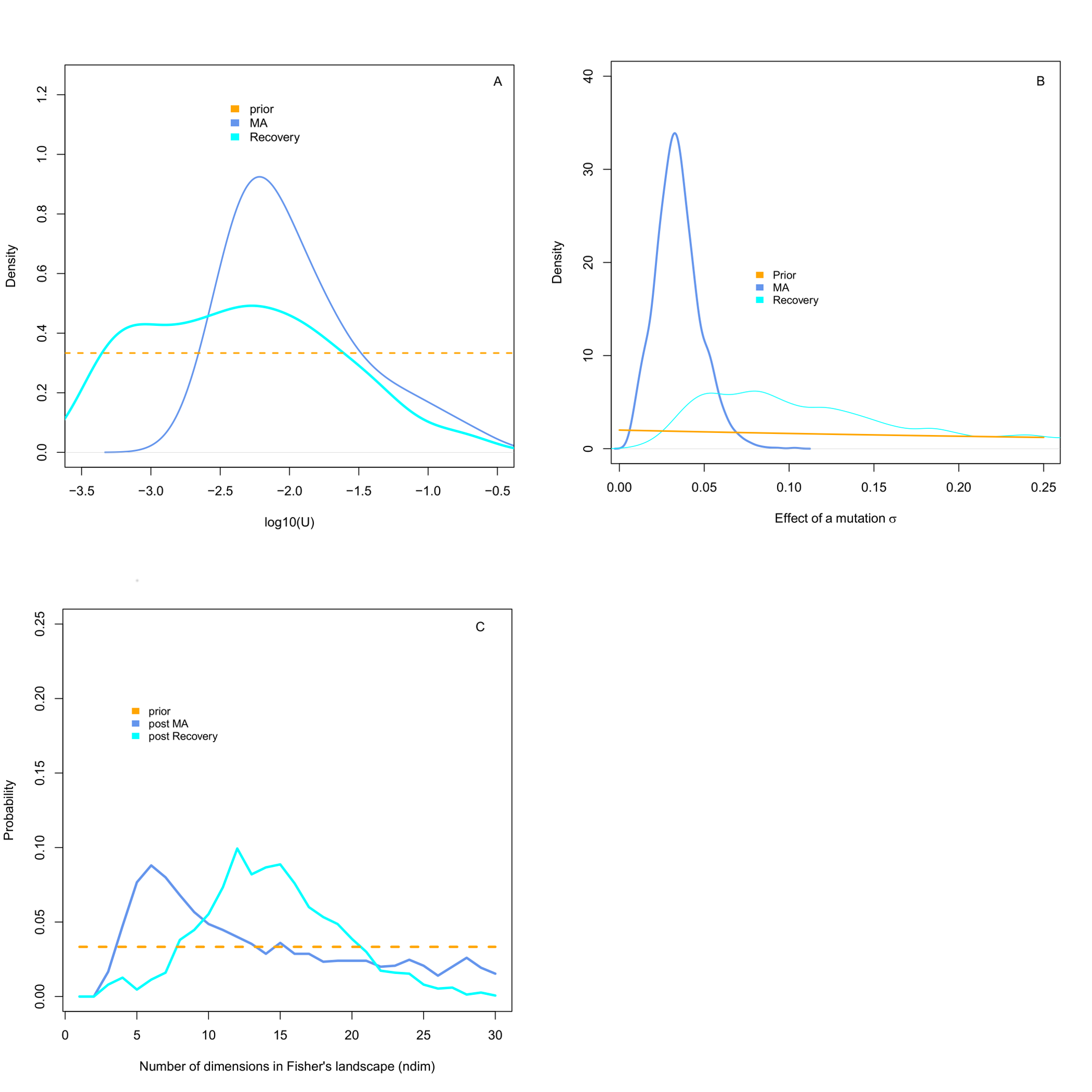

Supplement: Figure S5 — Posterior distributions obtained by fitting separately mutation accumulation and fitness recovery data sets. [file evo0068-0150-sd5.tif]
